# A web tool for finding gene candidates associated with experimentally induced arthritis in the rat

**DOI:** 10.1186/ar1700

**Published:** 2005-02-18

**Authors:** Lars Andersson, Greta Petersen, Per Johnson, Fredrik Ståhl

**Affiliations:** 1 Department of Cell and Molecular Biology – Genetics, Goteborg University, Sweden; 2 School of Health Sciences, University College of Borås, Borås, Sweden

## Abstract

Rat models are frequently used for finding genes contributing to the arthritis phenotype. In most studies, however, limitations in the number of animals result in a low resolution. As a result, the linkage between the autoimmune experimental arthritis phenotype and the genomic region, that is, the quantitative trait locus, can cover several hundred genes. The purpose of this work was to facilitate the search for candidate genes in such regions by introducing a web tool called Candidate Gene Capture (CGC) that takes advantage of free text data on gene function. The CGC tool was developed by combining genomic regions in the rat, associated with the autoimmune experimental arthritis phenotype, with rat/human gene homology data, and with descriptions of phenotypic gene effects and selected keywords. Each keyword was assigned a value, which was used for ranking genes based on their description of phenotypic gene effects. The application was implemented as a web-based tool and made public at . The CGC application ranks gene candidates for 37 rat genomic regions associated with autoimmune experimental arthritis phenotypes. To evaluate the CGC tool, the gene ranking in four regions was compared with an independent manual evaluation. In these sample tests, there was a full agreement between the manual ranking and the CGC ranking for the four highest-ranked genes in each test, except for one single gene. This indicates that the CGC tool creates a ranking very similar to that made by human inspection. The exceptional gene, which was ranked as a gene candidate by the CGC tool but not in the manual evaluation, was found to be closely associated with rheumatoid arthritis in additional literature studies. Genes ranked by the CGC tools as less likely gene candidates, as well as genes ranked low, were generally rated in a similar manner to those done manually. Thus, to find genes contributing to experimentally induced arthritis, we consider the CGC application to be a helpful tool in facilitating the evaluation of large amounts of textual information.

## Introduction

Rheumatoid arthritis (RA) is an autoimmune disease characterised by chronic inflammation of the joints. The prevalence of RA is 0.5 to 1% in many populations [1] and is about 2.5 times higher in women [2]. RA has a very complex genetic basis, and the combination of genetic and environmental causative factors makes it hard to study. The genetic contribution to RA susceptibility is estimated to be between 30% and 50%, of which the major histocompatibility complex accounts for about one-third [3].

Animal models provide a valuable tool for finding genes contributing to the susceptibility to and severity of RA. Rats are very useful for this purpose because autoimmune experimental arthritis phenotypes can be induced in susceptible strains by several agents, such as collagen, pristane, oil, streptococcal cell wall and even adjuvant alone [4-6]. Intercrosses of such susceptible rat strains with resistant strains are used for establishing linkage between genetic markers and quantitative traits distinguishing the arthritis phenotype. Statistically valid linkage between such genomic regions and measurements of quantitative traits are called quantitative trait loci (QTLs). More than 40 QTLs that regulate experimentally induced arthritis have been identified in different rat crosses [7]. Most of these QTLs are several megabases in size, containing many possible gene candidates. Several experimental strategies are used to narrow these regions, and these attempts almost always are combined with the retrieval of potential candidate genes found in different databases.

Information about RA and related genome data is available in several different forms, from raw data to descriptive text. One important difference between raw data and data based on human evaluation is that human evaluation often yields an interpretation that gives meaning to the data. Thus, human considerations bring an added value to genome data, which makes textual description an important source for investigating gene function. However, the amount of free text about RA is growing very fast, so there is an increasing need for developing a tool to help scientists distinguish relevant information from background noise. To facilitate this kind of data mining, we have created a tool, the Candidate Gene Capture (CGC) application, that makes keyword-based searches on textual information for genes situated within selected human chromosomal intervals that are homologous to a given rat QTL. Depending on the connection to RA, the keywords are allocated different values. The values for all matching keywords are summarised for each gene, the final values indicating which genes might be good candidates for contributing to the arthritis phenotype. When evaluated, this approach produces similar rankings to those done manually. In addition, this approach also manages to predict several candidate genes that are already established in the literature. Thus, the CGC application is a helpful tool for finding candidate genes associated with experimentally induced arthritis in rat.

## Materials and methods

The focus of this work is the development of a web-based tool that facilitates the identification of potential gene candidates that contribute to experimentally induced autoimmune arthritis. The application, called CGC, was created by combining QTL regions in rat with human gene homology data, descriptions of phenotypic gene effects and selected keywords.

### QTL data

Data describing 37 experimentally induced autoimmune arthritis QTLs in rat were obtained from the RatMap database [7]. These data were originally collected from experimentally induced inflammatory arthritis in rat strains susceptible to the following inducing agents: pristane, collagen, streptococcal cell wall, oil or adjuvant alone. Accordingly, the resulting QTLs are named Pristane-induced arthritis (Pia), Collagen-induced arthritis (Cia), Streptococcal cell wall-induced arthritis (Scwia), Oil-induced arthritis (Oia) and Adjuvant-induced arthritis (Aia).

The QTL data retrieved from RatMap include the locus symbol, a QTL description, the chromosomal position and flanking markers defining the borders of the QTL. The range of each QTL was based on the LOD score thresholds suggested in the corresponding papers. These data were stored in a MySQL table labelled 'QTL'.

### Gene homology data

Human gene data were assembled primarily from National Centre for Biotechnology Information (NCBI) [8] and the University of California Santa Cruz genome browser [9]. The genome information from NCBI consisted of official gene symbol, chromosome number, Locus Link ID, Online Mendelian Inheritance in Man (OMIM) ID, human Genome Database (GDB) accession ID and Refseq ID. Sequence positions were obtained exclusively from the University of California Santa Cruz genome browser, comprising transcript start/stop, codon start/stop, exon start/stop and number of exons in each gene. From this set of data, a table of human genes ordered by codon start was generated and labelled 'HsRn'.

To find orthologous gene pairs between rat and human, 1,464 chromosomally localised rat genes were obtained from RatMap. About 1,000 of these genes had a known homologous gene mapped in human. The orthologous rat/human gene pairs were characterised by the human data already present in table 'HsRn' together with the official rat gene symbol, rat chromosome number and RatMap ID.

Two flanking markers define each QTL used in this study. To find a human sequence homologous to a rat QTL region, an integrated linkage map containing rat genes and polymorphic DNA markers was used . For each QTL a pair of rat genes (obtained from the integrated linkage map) that were localised at, or close to, the two markers flanking the QTL and orthologous to human genes, was selected. The human chromosomal interval defined by these two orthologous genes was expected to contain a sequence homologous to the rat QTL. Because the homologous QTL interval often contained segments from more than one human chromosome, all orthologous rat/human gene pairs within each QTL were used to find smaller human chromosomal segments to comprise the total list of human genes confined within the homologous region. Information on rat and human gene symbols, chromosomal positions and codon start for all genes included in the homologous interval (obtained from table 'HsRn') was stored in QTL-specific tables labelled with the same symbol as the corresponding QTL.

### Downloading gene function data

The OMIM database [10] contains a comprehensive record of gene function and clinical data, which was used as a source for keyword querying in the CGC application. For each human gene within the selected intervals, gene function information was downloaded from OMIM and stored in a table labelled 'OMIMdata'.

### Selecting keywords and running the application

The querying process in this application is divided into four steps: finding a QTL of interest, displaying the rat/human homologous QTL region, selecting and ranking keywords, and searching OMIM text for selected keywords.

#### Finding a QTL of interest

The first step in finding candidate genes for a specific QTL is to choose a QTL of interest. To make this possible, we simply made the QTL database table directly available through a web interface. In this way, the user can access all QTLs in our database by searching for the locus symbol, the chromosome number and/or a descriptive text. The resulting QTLs are presented, together with a brief description obtained from the QTL table.

#### Displaying the rat/human homologous QTL region

Next, the user can select the preferred QTL. The resulting web page presents all rat/human gene pairs within the chosen rat QTL region, together with all human genes in the homologous human genomic region that are found in OMIM. These data are obtained from the corresponding 'QTL-specific' table.

Thus, all rat genes within a selected QTL and all genes within the homologous human genomic region are displayed. Because the human genome is better characterised than the rat genome, more human genes are usually displayed.

#### Selecting and ranking of keywords

For all arthritis QTLs a total of 49 default keywords were chosen. Most keywords were obtained by selecting all terms found directly under the MeSH (Medical Subject Headings) terms 'autoimmune diseases' and 'rheumatoid arthritis' in the PubMed MeSH-term database [11]. Some of these terms were truncated to optimise the querying process. In addition, a set of keywords frequently used in arthritis-related literature was added to the default keyword list.

To estimate the relative importance of the default keywords in relation to arthritis, each keyword was given a value depending on its relevance to arthritis. This relevance index was calculated as the number of PubMed abstracts containing both the keyword and the word 'arthritis' divided by the total number of abstracts containing the keyword alone. The relevance indices were multiplied by 100 to generate the final keyword values as percentages.

The application also allows the user to add up to 10 keywords of his or her own choice, and the corresponding keyword values are automatically generated on the basis of the same principle as for the default keyword values. Optionally, the user can overrule all keyword values, including the default ones.

#### Searching OMIM text for selected keywords

When searching a QTL for all the default keywords, alternatively deselecting unwanted ones and/or adding new ones, the keyword values for all keywords found within each OMIM text (locally stored in the table 'OMIMdata') will be summarised. To take advantage of the large amount of knowledge concerning the human genome, records in OMIM for all genes within the human homologous segment are used in the search, including genes not present in the rat gene list. For each gene, the total sum of all keyword values will be displayed, which indicates its relevance as a candidate gene. Each keyword is only counted once, independently of the number of times it occurs within a given OMIM text.

## Results

In the CGC application presented in this paper, all known rat genes within a selected QTL, along with all human genes within the homologous interval, are retrieved and displayed from a table that has the same name as the selected QTL. A list with an array of 49 selectable arthritis related keywords is presented together with their respective keyword values. Up to 10 additional keywords can be added and their keyword values are automatically calculated. When performing a search, the textual information for each human gene stored in the table 'OMIMdata' is scanned for all selected keywords. The genes and all keywords found in the accompanying text are displayed, together with the sum of all matching keyword values.

To estimate whether the CGC application was able to rank candidate genes in fashion similar to human evaluations, gene descriptions for four randomly selected QTL regions (*Cia4*, *Cia10*, *Cia14 *and *Cia17*) were surveyed manually. For all genes within the selected QTL regions, we compared the outcome of the CGC gene ranking with our own manual evaluation of each OMIM text. The manual rating was made without knowledge of the CGC ranking. To put the application and the manual inspection at a similar level, we tried to base our evaluation on the written OMIM texts only, without taking other information into account. In the manual inspection the OMIM texts were divided into five different classes: (1) obvious gene candidate, (2) likely gene candidate, (3) possible gene candidate, (4) unlikely gene candidate and (5) gene without relevance.

In addition, the genes that were ranked as high by the CGC application were further scrutinised in an extensive analysis of related papers not found in the OMIM reference lists. Finally, the *NCF1 *gene was studied in detail.

### Cia4

In total, 12 genes were ranked by the CGC tool. *IFNG *was rated as the top candidate by the CGC application and it was also considered to be the most appropriate gene candidate for collagen-induced arthritis within this QTL according to the manual inspection. *IL22 *was considered the next highest gene candidate both by the CGC application and the manual inspection.

#### *IFNG *(interferon-γ), CGC points 291.1, CGC ranking 1, manual rating 1

*IFNG *was identified by the CGC application on the basis of 10 different keywords: 'rheumatoid', 'HLA', 'sjogren', 'T cell', 'mhc', 'lymphocyte', 'antigen', 'cytokine', 'arthritis' and 'infecti'. *IFNG *has been shown to be closely associated with RA. In a study of 99 patients with RA of different severity, susceptibility to, and severity of, RA was shown to be related to a microsatellite polymorphism within the first intron of the gene encoding interferon-γ [12].

#### *IL22 *(interleukin-22), CGC points 14.1, CGC ranking 2, manual rating 2

*IL22 *was selected by the keywords 'inflam', 'T cell', 'lymphocyte' and 'cytokine'. *IL22 *activates three different *STAT *genes: *STAT1*, *STAT3 *and *STAT5 *[13]. RA synovial fibroblasts are relatively resistant to apoptosis and exhibit dysregulated growth. Retrovirus-mediated gene transfer of dominant-negative mutant *STAT3 *genes blocks the endogenous *STAT3 *expression in synovial fibroblasts from patients with RA, leading to failure of growth in the cell culture and apoptosis [14].

A middle group of two genes was selected with the CGC application: *MYC *(CGC points 10.9, CGC ranking 3, manual rating 3) and *HMGIC *(CGC points 10.5, CGC ranking 4, manual rating 4).

### Cia10

In total, 35 genes were ranked by the CGC tool. *RPL7 *and *NKFB1 *were ranked as the two top candidates by the CGC application. These two genes were also manually considered to be the most appropriate gene candidates for collagen-induced arthritis within this QTL.

#### *NFKB1 *(nuclear factor κB 1), CGC points 219.7, CGC ranking 1, manual rating 1

The very high point that *NFKB1 *obtained from the keyword query was in part due to the word 'arthritis' appearing in the corresponding OMIM text. Twelve other keywords were also found to be making a substantial contribution. According to the OMIM record, *NFKB1 *is a very strong gene candidate because the inappropriate activation of *NKFB1 *is known to be linked to inflammatory events associated with autoimmune arthritis [15].

#### *RPL7 *(ribosomal protein L7), CGC points 37.3, CGC ranking 2, manual rating 1

The *RPL7 *gene was rated second by the CGC application mainly because of the keywords 'autoimmune', 'lupus' and 'erythematosus'. The RPL7 protein is reported to be a major autoantigen in systemic autoimmune arthritis [16].

A middle group of five genes was rated as relatively high by the CGC application: *COL6A3 *(CGC points 24.2, CGC ranking 3, manual rating 3), *CSF1 *(CGC points 17.4, CGC ranking 4, manual rating 3), *EDG1 *(CGC points 12.5, CGC ranking 5, manual rating 5), *VCAM1 *(CGC points 11.3, CGC ranking 6, manual rating 2) and *PAPSS1 *(CGC points 9.3, CGC ranking 7, manual rating 3). Among these genes, *CSF1 *is a possible gene candidate because recent studies have shown that synovial tissue in RA joints secretes *CSF1 *together with several other cytokines, which increases the osteoclast activity [17]. *VCAM1 *might also be a potential gene candidate because it is expressed in endothelial cells of the blood vessels, facilitating the adhesion of leucocytes [18]. *EDG1 *was a false prediction because the term 'HLA' matched an author (**Hla **T. Maciag T. J Biol Chem 1990;265:9308-13) and the term 'T cell' matched 'mutan**t cell**'.

### Cia14

In total, 16 genes were ranked by the CGC tool. The two top ranked genes according to the CGC application (*IL15 *and *HMOX1 *) were also the highest-rated genes in the manual inspection.

#### *IL15 *(interleukin-15), CGC points 27.3, CGC ranking 1, manual rating 1

*IL15 *was ranked in first place by the CGC application. In the corresponding OMIM text, *IL15 *is associated with the keywords 'autoimmun', 'inflam', 'T cell', 'lymphocyte', 'antigen', 'cytokine' and 'infecti', but not 'arthritis'. In a recent paper it was shown that increased serum levels of *IL15 *are found in patients with long-term RA [19].

#### *HMOX1 *(haem oxidase 1), CGC points 13.5, CGC ranking 2, manual rating 1

*HMOX1 *was ranked second by the CGC application with the keywords 'anemia', 'hemolytic', 'inflam' and 'T cell'. *HMOX1 *has been shown to be involved in the treatment of RA with gold(I)-containing compounds. Gold(I) drugs selectively activate a transcription factor (Nrf2/small Maf heterodimer), which induces the transcription of anti-oxidative stress genes, including *HMOX1*, and inhibits inflammation [20].

A middle group of four genes were rated as relatively high by the CGC application: *ITK *(CGC points 9.7, CGC ranking 3, manual rating 2), *NFATC3 *(CGC points 9.7, CGC ranking 3, manual rating 3), *AARS *(CGC points 9.2, CGC ranking 5, manual rating 3) and *KARS *(CGC points 9.2, CGC ranking 5, manual rating 3).

### Cia17

In total, 30 genes were ranked by the CGC tool (only one member of the *PCDH *gene family was included). In the manual inspection, no 'obvious' candidate gene was found. However, four genes were considered to be 'likely' gene candidates. One of these, *CD74*, also received the highest keyword sum in the CGC application. Another gene among the likely gene candidates, *SLC26A2*, was ranked second by the CGC application.

#### *CD74*, CGC points 27.7, CGC ranking 1, manual rating 3

The *CD74 *gene was ranked in first place by the CGC application because of results from six different keywords: 'antigen', 'HLA', 'immunoglobulin', 'T cell', 'MHC' and 'inflam'. In a recent paper by Leng and colleagues [21], not present in the OMIM text, *CD74 *is reported to be required for macrophage migration inhibitory factor (MIF)-induced activation of the extracellular signal-regulated kinase-1/2 mitogen-activated protein kinase cascade, cell proliferation, and prostaglandin E_2 _production. MIF is an upstream activator of monocytes/macrophages and is centrally involved in the pathogenesis of RA and other inflammatory conditions.

#### *SLC26A2 *(solute carrier family 26 member 2), CGC points 24.2, CGC ranking 2, manual rating 2

*SLC26A2 *was associated with the keyword 'joint'. *SLC26A2 *is an anion transporter responsible for four recessively inherited chondrodysplasias: multiple epiphyseal dysplasia (MED) [22], diastrophic dysplasia (DTD) [23], atelosteogenesis Type II (AO2) [24] and achondrogenesis type IB (ACG1B) [25]. However, although other forms of chondrodysplasias such as progressive pseudorheumatoid chondrodysplasia show symptoms similar to those of RA, no clear link between *SLC26A2 *and RA can be concluded.

A middle group of four genes were ranked in positions 3 to 6 by the CGC application: *NR3C1 *(CGC points 16.5, CGC ranking 3, manual rating 2), *SPINK5 *(CGC points 14.2, CGC ranking 4, manual rating 3), *IK *(CGC points 14.1, CGC ranking 5, manual rating 3) and *CD14 *(CGC points 12.8, CGC ranking 6, manual rating 2). Two of these genes might be related to RA. *NR3C1 *is significantly overexpressed in untreated patients with RA and in several clinical studies of inflammatory conditions, such as RA [26]. *CD14 *has been reported to be associated with significantly elevated serum levels in patients with RA [27,28].

### *NCF1 *(neutrophilic cytosolic factor 1)

The gene *NCF1 *is covered by both the *Cia12 *and *Pia4 *QTLs and was assigned a total point of 238.9 by the CGC application. This suggests that *NCF1 *is a strong gene candidate for RA. Indeed, *NCF1 *has been identified as a gene that has a naturally occurring polymorphism regulating arthritis severity in rats [29]. On looking at the OMIM text for *NCF1*, it is clear that most of the points come from the part of the text describing these particular findings. To evaluate the ability of the tool to predict genes that are reported to be related to the arthritis phenotype, the OMIM text was used in the form in which it existed before *NCF1 *was shown to be associated with arthritis; that is, the part of the OMIM text describing the association between *NCF1 *and arthritis was deleted before running the application. The resulting keyword sum was, as expected, much lower, with a total point of 10.8. However, these points were still sufficient to rank *NCF1 *as the top candidate of *Cia12 *and *Pia4 *. Recently, the gene *GUSB *was updated at OMIM, resulting in a total point of 30.7.

## Discussion

A common feature of many genetically orientated RA studies is to find genes responsible for, or contributing to, one or several RA-related phenotypes. Typically, a genomic region might be known to be associated with a phenotype, but still there are usually many genes within such a region that might be possible candidates. Specifically, when employing QTL analysis in rats, selecting gene candidates has become a recurrent part of the data analysis. An important part of the search for candidate genes is checking the available bioinformatic resources; most often the written information describing gene function is very informative. The aim of this study was to facilitate this data mining by generating a web-based tool called Candidate Gene Capture (CGC), whose purpose is to identify potential candidate genes associated with experimentally induced arthritis phenotypes in rats.

In brief, the CGC application makes it possible to retrieve a large number of QTL regions previously described in the literature. For each rat QTL, the homologous genomic region in humans is automatically displayed. All genes included in the corresponding human genomic interval can be queried for up to 49 default keywords and up to 10 keywords selected by the user. Each keyword is given a value based on an algorithm that estimates how closely related a keyword is to the term 'arthritis' according to their simultaneous occurrence in PubMed abstracts. OMIM records for human genes in a selected genomic region are ranked by their total keyword values; that is, the sum of the values for all keywords that hit a record. The higher the total keyword sum is, the more likely it is to be a gene candidate. The application can be accessed from the RatMap home page [7] or directly at .

### Comparison of manual evaluation with CGC ranking

To estimate the ability of the CGC application to rank candidate genes in a fashion similar to human evaluation, an independent manual inspection was made. Four randomly selected collagen-induced arthritis QTLs were used (*Cia4*, *Cia10*, *Cia14 *and *Cia17 *). The OMIM records used in the CGC prediction were surveyed manually and rated on a scale from 1 to 5. Comparing the manual and CGC ratings, it was found that the two highest-ranked candidate genes in the CGC application for all QTLs studied were rated as high in the manual evaluation, with the exception of one gene, *CD74 *in *Cia17 *. However, *CD74 *turned out to be a very likely gene candidate when additional literature was surveyed (see below).

In an extended literature search for the two highest CGC-ranked genes of *Cia4*, *Cia10*, *Cia14 *and *Cia17*, it was confirmed that seven of eight genes were clearly associated with RA. Literature not covered by the OMIM reference lists revealed that three of these genes (*IL5*, *CD74 *and *HMOX1 *) had a strong association with RA. Many different keywords fitted each of the OMIM records associated with these three genes. Although none of these keywords had a very high keyword value (ranging from 1.6 to 9.7), the resulting keyword sums (*IL15*, 27.3; *CD74*, 22.3; *HMOX1*, 13.5) still clearly diverged from the keyword sums of other genes within the same QTLs. Thus, the CGC application is able to predict candidate genes from OMIM records even though the association with RA is not explicitly mentioned in the text.

In addition to the two highest-ranked genes in the four QTLs evaluated, we also designated a middle group of candidate genes that were ranked in positions 3 to 6 by the CGC application (except for *Cia4*, in which the middle group comprised genes ranked in positions 3 and 4). The remaining genes for each investigated QTL formed a separate group (the low group). Comparing the mean values of the CGC ranking with the manual ratings for these three groups (the two highest, the middle group and the low group), a general agreement was found in the ranking of candidate genes (Table 1). The only exception was the relatively low manually rated 'best two' group for *Cia17*, which is fully explained by the low manual rating of *CD74 *. As described above, on closer inspection the manual rating of *CD74 *turned out to be too cautious.

Finally, gene records without any keyword hits at all were not found to be associated with RA in the manual inspection.

Thus, when the CGC prediction is compared with manual inspection, the conclusion is that the application makes a reliable evaluation of the OMIM records for the four QTLs studied in detail. For three genes (*IL5*, *CD74 *and *HMOX1 *) the CGC application estimated the gene records as being more interesting than the manual inspection, an estimation confirmed by recent papers not yet included in the OMIM reference list. This shows that the CGC application is a very helpful tool for finding gene candidates contributing to RA. Furthermore, the CGC application also seems to follow our manual interpretation for genes that might be of interest (referred to as the 'middle group') as well as for genes with no evident connection to RA.

### Keywords

No clear-cut connection can be made between the absolute sum of keyword values and the relevance of candidate genes. However, our evaluation of the four Cia QTLs implies that the ranking of the genes within each QTL based on the keyword sums provides a good prediction of the best candidate genes. For example, in QTL region *Cia12*, *NCF1 *has been shown by Olofsson and colleagues to be involved in the regulation of arthritis severity in rats [29]. As expected, *NCF1 *also obtains a very high keyword sum (225.6), mainly because of the description of Olofsson's findings in the OMIM text. When this description is excluded from the OMIM record, the *NCF1 *keyword sum decreases to 10.8. This still made *NCF1 *the highest-ranked gene in this QTL region. As exemplified above, the CGC application is able to find candidate genes even though their relatedness to RA is not explicitly mentioned in the text investigated. In the paper describing Olofsson's findings, the authors stated that they found the candidate gene approach distracting, even though they were facing a region that contained a small set of genes. This could very well be so, but when analysing the genes within a QTL it seems reasonable to start with the most likely candidate genes rather than with randomly picked ones, especially if the region contains a large number of genes. The CGC application makes an unbiased evaluation of genes within a region, indicating which are the most favourable ones to start analysing. Looking at the *NCF1 *example retrospectively, CGC would in fact have suggested *NCF1 *as the most probable candidate gene, although this might be a fortunate case.

Among the selected keywords, occasionally there were a few that gave false positives. One example is the word 'joint' (point 24.2), which at times referred to other terms, such as 'joint maximum LOD score'. For example, this caused the gene *KEL *to be ranked highest (28.7) for the *Aia2 *QTL. Another example is 'T cell' (points 2.8), which can produce results such as mutan**t cell **or tha**t cell**, as found in the OMIM record for *EDG1 *(*Cia10 *). In addition, it was found that some keywords can be misinterpreted as author names. *EDG1*, for example, was falsely predicted as a candidate gene partly because the term 'HLA' matched an author (**Hla **T. Maciag T. J Biol Chem 1990;265:9308-13).

Forty-nine keywords were selected, based on PubMed MeSH terms and other terms frequently found in the literature on RA. However, this might not be a completely exhaustive set of keywords and a user of the CGC tool might want to extend or exchange parts of this keyword list. To make this possible, the user can add up to 10 keywords of his or her own and can automatically obtain the corresponding keyword values calculated. These keywords can be used alone or together with the whole or parts of the default keyword list. It should be emphasised that there is really no harm in using a large number of keywords, because irrelevant keywords, such as 'and' or 'is', will get almost no keyword values, thus not disturbing the selecting process. In addition, the user is allowed to overrule all keyword values if preferred and enter values of his or her own choice.

### Comparison with related databases

To our knowledge there are three databases other than CGC that address the problem of finding candidate genes for complex disorders.

GeneSeeker is a web-based tool that permits the user to search different databases simultaneously, given a known human genetic location and an expression or phenotypic pattern(s) [30]. Moreover, data from syntenic regions in mouse can be included in the queries. The tool is a general instrument that has its strength in the range of databases covered. However, GeneSeeker has no means for prioritizing between the genes retrieved. Because the CGC tool is specifically adapted for arthritis models, much more keywords relevant to this phenotype are available here although both applications permit the user to enter his or her own keywords.

POCUS (Prioritizing Of Candidate genes Using Statistics) is an application that rates genes on the basis of their similarity to a set of genes generally considered to be associated with a given complex trait [31]. The similarity is quantified by measuring the number of functional annotations (Gene Onthology terms or InterPro domain ID) and/or expression pattern terms and IDs in common (Unigene or NCBI). Although POCUS prioritizes between the gene candidates, the strategy is different from that used for CGC. The genes associated with a given trait are not restricted to a specific genomic region. However, the authors claim that the application might be extended to work in such a way. POCUS is not a web-based tool but can be downloaded.

G2D (candidate Genes To inherited Diseases) is another database accessible from the web [32]. G2D is built on a strategy resembling that of CGC. In brief, chemical terms have here been given scores calculated in a similar fashion to that in CGC; that is, the simultaneous occurrence of chemical terms (MeSH-C) and pathological conditions (MeSH-D) in PubMed. For a given disease several pathological conditions were selected on the basis of a set of representative papers. These pathological conditions were then related to functional descriptions (Gene Ontology terms) by using RefSeq annotations (RefSeq-NCBI) as mediating links, and the degree of relatedness were represented by 'GO-scores'. A gene can be related to a given disease by calculating the average GO-score annotated for that gene. In many ways this approach resembles that described in this paper, although G2D depends on Gene Onthology terms instead of a full text. Moreover, G2D uses the mean GO-score for rating genes rather than calculating the sum. As a consequence, a gene with a GO-score based on just a single Gene Ontology term is rated higher than a gene that is annotated for the same term together with additional Gene Ontology terms with lower scores. Furthermore, in contrast to CGC, the GD2 database is a static database in which no data input from the user is possible, and at present no information on RA is available.

### Future developments

As our next step we plan to evolve the CGC application to include other text-based resources, such as PubMed abstracts, Swiss-Prot descriptions and, as a complement, Gene Ontology terms. In addition, we are currently extending the CGC tool to include rat QTLs for metabolic disorders, mainly focused on diabetes mellitus type II. The long-term goal is that the CGC tool will be able to predict candidate genes for any given type of rat QTL, such as multiple sclerosis, blood pressure or obesity. The strategy used in CGC could also be applied on QTLs in other species, such as mouse or human.

## Conclusion

We conclude that the excellent agreement between our manual evaluation and the rankings made by the CGC application for the four different QTLs tested (*Cia4*, *Cia10*, *Cia14 *and *Cia17 *), as well as the prediction of the *NCF1 *gene, clearly show that this tool makes very reliable predictions. Consequently, we believe that the CGC tool can be of great use in facilitating the finding of gene candidates related to the arthritis phenotype.

## Abbreviations

Aia = Adjuvant-induced arthritis; CGC = Candidate Gene Capture; Cia = Collagen-induced arthritis; NCBI = National Centre for Biotechnology Information; OMIM = Online Mendelian Inheritance in Man; Pia = Pristane-induced arthritis; QTL = quantitative trait locus; RA = rheumatoid arthritis.

## Competing interests

The author(s) declare that they have no competing interests.

## Authors' contributions

LA performed the programming of the CGC application, contributed original ideas on assigning keyword values and drafted the manuscript. GP created the rat/human comparative database, implemented it in the CGC application and drafted the manuscript. PJ had main responsibility for all supporting functions of the application and was involved in the theoretical basis of the work. FS supervised the project, contributed with original ideas and took full part in the preparation of the manuscript. All authors read and approved the final manuscript.

## Figures and Tables

**Table 1:** Comparison between manual evaluation and Candidate Gene Capture (CGC) rating

	Best two		Middle group		Low group	
QTL	CGC	Manual	CGC	Manual	CGC	Manual
*Cia4*	152.6	1.5	10.7	3.5	2.3	3.9
*Cia10*	128.5	1.0	14.9	2.0	3.6	3.9
*Cia14*	20.4	1.0	9.5	2.7	4.6	3.4
*Cia17*	26.0	2.5	14.4	2.5	4.9	3.8

Mean values of keyword sums and manual ratings for genes in three groups are shown, on the basis of their ranking by the CGC application. QTL, quantitative trait locus.
